# Effect of extrusion on energy and nutrient digestibility of lentil-based diets containing either supplemental plant or animal protein fed to growing pigs

**DOI:** 10.1093/tas/txae017

**Published:** 2024-02-09

**Authors:** Joaquin Sanchez-Zannatta, Li Fang Wang, Eduardo Beltranena, Ruurd T Zijlstra

**Affiliations:** Department of Agricultural, Food and Nutritional Science, University of Alberta, Edmonton, AB, Canada T6G 2P5; Department of Agricultural, Food and Nutritional Science, University of Alberta, Edmonton, AB, Canada T6G 2P5; Department of Agricultural, Food and Nutritional Science, University of Alberta, Edmonton, AB, Canada T6G 2P5; Department of Agricultural, Food and Nutritional Science, University of Alberta, Edmonton, AB, Canada T6G 2P5

**Keywords:** digestibility, extrusion, fish meal, lentil, pig, soybean meal

## Abstract

Non-food grade and excess lentil grain production may be included in swine feeds to provide starch and protein and reduce feed cost. Extrusion processing may increase energy and nutrient digestibility of lentil-based diets containing either supplemental plant or animal protein sources. Therefore, the apparent ileal digestibility (**AID**) of crude protein (**CP**) and amino acids (**AA**), apparent total tract digestibility (**ATTD**) of gross energy (**GE**), and digestible energy (**DE**) value of lentil-based diets were assessed in growing pigs. Two diets were formulated to provide 2.4 Mcal net energy (**NE**)/kg and 4.35 g standardized ileal digestible lysine/Mcal NE: (1) soybean meal (**SBM**) diet, containing 50% lentil, 31% wheat, and 12.8% SBM; and (2) fish meal (**FM**) diet, containing 40% lentil, 45% wheat, and 10% FM. Following mixing, each diet batch was divided into two parts: one part remained as mash, whereas the other part was extruded using a single-screw extruder (400 rpm, 250 kg/h). Eight ileal-cannulated barrows (32.3 ± 1.5 kg) were fed the four diets at 2.8 times maintenance DE requirement (110 kcal per kg of body weight^0.75^) for four 9-d periods in a double 4 × 4 Latin square to achieve 8 observations per diet. Data were analyzed as a 2 × 2 factorial arrangement including protein source, post-mixing processing, and their interaction as fixed effects. The lentil sample contained 32.3% starch, 24.4% CP, 9.3% total dietary fiber, and 1.7 mg/g of trypsin inhibitor activity on as is-basis. Interactions between dietary protein source and post-mixing processing were not observed. Feeding FM diets resulted in greater (*P* < 0.05) AID of dry matter (**DM**), GE, and most AA, and ATTD of CP, but lower apparent hindgut fermentation of DM and GE than SBM diets. Extrusion increased (*P* < 0.05) the ATTD of GE and DE value of diets. The AID of CP and AA was 3.2 and 4.7%-units greater (*P* < 0.05), respectively, for the extruded than mash diets. In conclusion, feeding FM diets resulted in greater ileal digestibility of DM, GE, and AA than SBM diets. Extrusion increased the AID of CP and most AA, and DE value of lentil-based diets containing either supplemental plant protein or animal-protein, indicating that extrusion can increase the energy and protein value of plant-based diets fed to pigs.

## Teaser

Extrusion can increase the amino acid digestibility and energy value of diets based on lentil grain that contains either supplemental plant protein or animal protein in growing pigs.

## Introduction

Elevated global prices of cereal grains and protein sources have increased feed cost (OECD/FAO, 2022); thus, alternative feedstuffs are increasingly used for animal feeding to support the food demands of the growing world population (Zijlstra and Beltranena, 2022). Lentil (*Lens culinaris* ssp. *culinaris*) is a pulse grain mainly produced for human consumption. Starch and protein are the major constituents of lentil grain that serve as an important source of nutrition for humans. Annually, around 4.5 million tonnes (**Mt**) of lentil grain is produced globally (Chelladurai and Erkinbaev, 2020). In Canada, lentil is after field pea the second pulse crop with 2.4 Mt produced annually of which 2.1 Mt are exported (AAFC, 2023), but non-food grade or excess production of lentil grain is readily available for animal feeding. Including lentil or other pulses as crops in cereal and oilseed rotations provides rhizobia-mediated N fixation to the soil, helps break pest and disease cycles, and reduces the carbon and nitrogen footprint of producing plant-based protein (Warne et al., 2019).

Lentil grain contains more digestible amino acids (**AA**) than wheat or corn and has a greater energy value than soybean meal (**SBM**) or fish meal (**FM**) (NRC, 2012). However, lentil has a relatively high content of dietary fiber that may reduce nutrient digestibility and energy value in diets for pigs (Lindberg, 2014). Lentil had lower apparent ileal digestibility (**AID**) and standardized ileal digestibility (**SID**) of AA than SBM in growing pigs (Woyengo et al., 2014). Thus, the addition of supplemental protein or AA to plant-based diets is often necessary to provide a balanced dietary amino acid profile for pigs and dogs (Quilliam et al., 2021). Animal protein such as FM is used as source of highly digestible protein, rich in indispensable AA, such as lysine and methionine (Jones et al., 2010). However, the high cost of FM and demand for sustainable feed has increased the interest in plant-based dietary protein sources (Van Zanten et al., 2019). Feed processing methods such as extrusion that is used commonly to produce pet food and aquaculture feed may increase nutrient utilization of plant-based diets (Romarheim et al., 2005; Tran et al., 2008). Extrusion conditions may gelatinize starch and thereby increase energy digestibility and may denature proteins and thereby increase protein digestibility, whereas excessive heating may damage protein (Sun et al., 2006; Tran et al., 2008). However, extrusion of complete diets fed to pigs to enhance nutrient utilization has rarely been reported. Information is also lacking on the effect of extrusion between diets containing either supplemental plant or animal protein in pigs.

The null hypothesis of the present study was that the digestibility of nutrients and energy would not differ among growing pigs fed mash or extruded lentil-based diets containing supplemental plant protein (SBM) or animal protein (FM). The objective of the study was to compare the AID and apparent total tract digestibility (**ATTD**) of crude protein (**CP**), AA, and gross energy (**GE**), and the calculated digestible energy (**DE**) value of mash or extruded lentil-based diets containing either supplemental plant or animal protein in ileal-cannulated growing pigs.

## MATERIALS AND METHODS

Animal use was approved, and procedures were reviewed by the University of Alberta Animal Care and Use Committee for Livestock, and followed principles established by the Canadian Council on Animal Care (CCAC, 2009). The study was conducted at the Swine Research and Technology Centre, University of Alberta (Edmonton, AB, Canada).

### Diets, Animals, and Experimental Procedures

Two diets containing either plant or supplemental animal protein sources were formulated. The diet with supplemental plant protein was based on lentil, wheat grain, and SBM; and the diet with supplemental animal protein was based on wheat grain, lentil, and FM (Table 1). Diets were formulated to provide 2.4 Mcal NE/kg and 4.35 g standardized ileal digestible Lys/Mcal NE. Diets were supplemented with vitamins and minerals to exceed mineral and vitamin requirements of pigs (NRC, 2012). Diets contained 0.50% titanium dioxide as an indigestible marker. The red lentil (*Lens culinaris*) was sourced from CorNine Commodities (Lacombe, AB, Canada) and the FM was a commercial source of menhaden FM (Omega Protein, Reedville, VA). Before mixing the diets, lentil and wheat grain were ground using a hammer mill through a 2.78-mm screen (Jacobson model 5550-113-01, Carter Day International, Minneapolis, MN). Diets were mixed in a horizontal paddle mixer (model SPC2748, Marion Mixers Inc., Marion, IA). After mixing, diets were divided into two parts: one part was kept as mash (SBM and FM diets) and the other part was extruded using a single-screw extruder (model X-115; Wenger, Sabetha, KS) at the Agri-Food Discovery Place, University of Alberta (Edmonton, AB, Canada). Screw speed was maintained at 400 rpm at a constant feed rate of 250 kg/h. Extrusion temperature was set at 95, 100, and 105°C for zones 1, 2 and 3, respectively, and 114°C for zones 4 and 5. The extruded diets were subsequently ground through a roller mill to achieve a mean particle size between 600 and 700 µm.

**Table 1.** Ingredient composition of experimental diets containing supplemental plant or animal protein with or without extrusion^1^

	Supplemental plant protein		Supplemental animal protein	
Ingredient, %	Mash	Extruded	Mash	Extruded
Lentil^2^	50.00	50.00	40.00	40.00
Wheat, Hard Red Spring	31.02	31.02	45.03	45.03
Soybean meal	12.80	12.80	—	—
Fish meal^3^	—	—	10.00	10.00
Canola oil	1.82	1.82	1.00	1.00
Limestone	1.37	1.37	1.30	1.30
Mono-/di-calcium phosphate	0.80	0.80	0.61	0.61
TiO_2_	0.50	0.50	0.50	0.50
Salt	0.50	0.50	0.50	0.50
Vitamin premix^4^	0.50	0.50	0.50	0.50
Trace mineral premix^5^	0.50	0.50	0.50	0.50
DL-Methionine	0.11	0.11	0.01	0.01
L-Lysine HCl	0.03	0.03	—	—
L-Tryptophan	—	—	0.01	0.01
L-Threonine	—	—	0.01	0.01
Choline chloride, 60%	0.05	0.05	0.05	0.05

^1^Diets were formulated to provide (as fed): 2.4 Mcal net energy (NE)/kg and 4.35 g standardized ileal digestible (SID) lysine/Mcal NE.

^2^Red lentil was sourced from CorNine Commodities (Lacombe, AB, Canada).

^3^Menhaden fish meal (Omega Protein, Reedville, VA).

^4^Supplied per kilogram of diet: 7,500 IU of vitamin A, 750 IU of vitamin D, 50 IU of vitamin E, 37.5 mg of niacin, 15 mg of pantothenic acid, 2.5 mg of folacin, 5 mg of riboﬂavin, 1.5 mg of pyridoxine, 2.5 mg of thiamine, 2000 mg of choline, 4 mg of vitamin K, 0.25 mg of biotin, and 0.02 mg of vitamin B_12_.

^5^Supplied per kilogram of diet: 125 mg of Zn as ZnSO_4_, 50 mg of Cu as CuSO_4_, 75 mg of Fe as FeSO_4_, 25 mg of Mn as MnSO_4_, 0.5 mg of I as Ca (IO_3_)_2_, and 0.3 mg of Se as Na_2_SeO_3_.

The 4 diets were fed to 8 cannulated pigs over four periods in a double 4 × 4 Latin square to achieve 8 observations per diet. Eight crossbred barrows (initial body weight [**BW**] 32.3 ± 1.5 kg; Duroc × Large White/Landrace F1; Genex Hybrid; Hypor, Regina, SK, Canada) were surgically fitted with a simple T-cannula at the distal ileum. Pigs were housed individually in metabolism pens (1.2 m wide, 1.4 m long, and 0.94 m high) in a temperature-controlled room with a photoperiod from 0700 to 1900 hours. Pens were equipped with a stainless-steel feeder attached to the front of the pen and a cup drinker, polyvinyl chloride walls with windows and plastic slatted flooring. Daily feed allowance was set to 2.8 times the maintenance requirement (2.8 × 110 kcal of DE/kg of BW^0.75^; NRC, 1998) fed in 2 equal meals at approximately 0800 and 1500 h. Each 9-d experimental period consisted of a 5-d acclimation to experimental diet, followed by a 2-d collection of feces and a 2-d collection of ileal digesta. Feces were collected continuously using plastic bags within a collection system that was glued to the skin around the anus (van Kleef et al., 1994). Digesta was collected continuously for 10 h from 0800 to 1600 hours using plastic bags (containing 15 mL of 5% formic acid) attached to the opened cannula barrel (Li et al., 1993). Collected feces and ileal digesta were pooled for each pig and period and frozen at−20 °C. At the end of the trial, specimens were thawed, homogenized, sub-sampled, and freeze-dried.

### Chemical Analyses

Lentil, SBM, FM, diets, and lyophilized ileal digesta and feces were ground using a centrifugal mill (model ZM 200; Retsch GmbH, Haan, Germany) through a 1-mm screen and analyzed for dry matter (**DM**; method 930.15; AOAC, 2006), CP (method 990.03; N × 6.25; AOAC, 2006), and GE using an adiabatic bomb calorimeter (model 5003; Ika-Werke, Staufen, Germany). Lentil, FM and diets were analyzed for ash (method 942.05; AOAC, 2006), crude fat (method 920.39A; AOAC, 2006), crude fiber (method 978.10; AOAC, 2006), acid detergent fiber (**ADF**) inclusive of residual ash (method 973.18; AOAC, 2006), neutral detergent fiber (**NDF**) assayed without heat-stable amylase and expressed inclusive of residual ash (Holst, 1973) and total dietary fiber (**TDF**), soluble and insoluble dietary fiber (method 991.43; AOAC, 2006). Lentil, SBM, and FM were analyzed for starch (assay kit STA-20; Sigma, St. Louis, MO), Ca (method 968.08; AOAC, 2006) and P (method 946.06; AOAC, 2006). The AA content in lentil, SBM, FM, diets, and digesta was analyzed by high-performance liquid chromatography (method 982.30E; AOAC, 2006), and chemically available lysine was analyzed by spectrophotometry (method 975.44; AOAC, 2006). Diets, feces, and digesta were analyzed for titanium dioxide by spectrophotometry (Myers et al., 2004). Lentil was analyzed for trypsin inhibitor activity (**TIA**) (method NEN-EN-ISO 14902:2001; Nederlands Normalisatie-Instituut [NEN, 2001)]) at Nutrilab (Giessen, NB, Netherlands).

### Calculations and Statistical Analyses

The AID and ATTD of nutrients and GE in diets were calculated using the indicator method with the following equation (Adeola, 2001):


$$\begin{array}{lll} & AID\;or\;ATTD=\; & \left(1-\frac{Marker\;in\;feed\;\times \;content\;of\;component\;in\;feces\;or\;digesta}{Marker\;in\;feces\;or\;digesta\;\times \;content\;of\;component\;in\;feed}\right)\times 100 \end{array}$$

The difference between ATTD and AID was considered as apparent hindgut fermentation (**AHF**). The DE values of diets were calculated by multiplying GE by its ATTD (Adeola, 2001).

Data were analyzed as a 2 × 2 factorial arrangement with supplemental protein source and post-mixing processing (extrusion versus mash) as factors using the GLIMMIX procedure of SAS with a normal distribution and the identity link function. The model included supplemental protein source, extrusion, and the supplemental protein source × extrusion interaction as fixed effects, whereas square, period nested in square, and pig nested in square were random effects. Diet fed in the previous period was tested as a covariate to determine if carryover effect existed. The normality and homogeneity of the residual of each variable were confirmed using the UNIVARIATE procedure of SAS with ‘Normal’ option and GLM procedure with ‘Hovtest = Levene’ option, respectively. Pig was the experimental unit for all analyses. To test the hypotheses, *P* < 0.05 was considered significant.

## RESULTS

Extrusion did not affect CP, TDF, NDF, and ADF but reduced crude fat content of diets by up to 50% (Table 2). The lentil sample contained 32.3% starch, 24.4% CP, 9.3% total dietary fiber, and 1.7 mg/g of trypsin inhibitor activity on an as-is basis (Table 3). Arginine, leucine, and lysine were the most abundant indispensable AA in the lentil sample, whereas the least abundant were tryptophan, methionine, and histidine. The FM sample contained 34, 45, 66, 36, and 19% more CP, lysine, methionine, threonine, and tryptophan than the SBM sample, respectively. The FM and SBM samples had a similar GE value.

**Table 2.** Analyzed nutrient content (as fed) of experimental diets containing supplemental plant or animal protein with or without extrusion

	Supplemental plant protein		Supplemental animal protein	
Item	Mash	Extruded	Mash	Extruded
Analyzed nutrient content, %				
Dry matter	90.15	90.80	89.93	91.71
Crude protein	22.42	22.35	23.22	22.76
Total dietary fiber	8.21	8.25	9.61	9.67
Insoluble dietary fiber	8.11	8.06	9.21	9.11
Soluble dietary fiber	< 0.10	< 0.10	0.10	0.10
Neutral detergent fiber	7.43	6.99	8.07	8.33
Acid detergent fiber	4.76	4.44	4.08	3.87
Crude fiber	2.95	3.13	2.94	2.82
Ash	5.35	5.67	6.61	7.25
Crude fat	2.93	1.71	2.64	1.32
Gross energy, Mcal/kg	4.01	4.03	3.95	4.00
Indispensable amino acids, %				
Arginine	1.43	1.47	1.41	1.43
Histidine	0.52	0.54	0.52	0.53
Isoleucine	0.94	0.94	0.93	0.95
Leucine	1.53	1.58	1.57	1.60
Lysine	1.27	1.25	1.33	1.34
Methionine	0.31	0.34	0.37	0.37
Phenylalanine	1.05	1.07	1.02	1.03
Threonine	0.76	0.77	0.79	0.82
Tryptophan	0.21	0.18	0.21	0.23
Valine	1.02	1.03	1.03	1.05
Dispensable amino acids, %				
Alanine	0.87	0.89	1.03	1.04
Aspartic acid	2.07	2.13	1.98	2.02
Cysteine	0.30	0.30	0.31	0.30
Glutamic acid	4.08	4.14	4.16	4.20
Glycine	0.88	0.91	1.15	1.14
Proline	1.17	1.16	1.25	1.27
Serine	0.87	0.92	0.86	0.89
Tyrosine	0.65	0.67	0.65	0.67

**Table 3.** Analyzed nutrient composition (as is) of lentil, fishmeal, and soybean meal included in experimental diets

Item, %	Lentil	Fish meal	Soybean meal
Dry matter	89.19	92.80	93.33
Starch	32.26	—	1.33
Crude protein	24.43	66.71	44.00
Total dietary fiber	9.27	<1.00	13.27
Insoluble dietary fiber	7.88	<1.00	12.89
Soluble dietary fiber	1.38	—	0.38
Neutral detergent fiber	8.42	4.18	8.02
Acid detergent fiber	5.38	2.48	4.43
Crude fiber	3.83	0.66	3.80
Ash	2.41	18.85	5.90
Crude fat	0.11	8.56	3.18
Calcium	0.07	5.20	0.26
Phosphorus	0.31	3.05	0.66
Gross energy, Mcal/kg	3.96	4.42	4.46
Indispensable amino acids			
Arginine	1.77	4.01	3.06
Histidine	0.59	1.52	1.15
Isoleucine	1.08	2.81	2.07
Leucine	1.75	4.61	3.36
Lysine	1.69	5.07	2.78
Methionine	0.19	1.82	0.62
Phenylalanine	1.20	2.50	2.21
Threonine	0.87	2.69	1.71
Tryptophan	0.13	0.72	0.58
Valine	1.19	3.18	2.25
Dispensable amino acids			
Alanine	1.00	4.01	1.90
Aspartic acid	2.65	5.89	4.88
Cysteine	0.26	0.58	0.67
Glutamic acid	3.74	8.48	7.95
Glycine	0.98	4.53	1.87
Proline	0.88	2.97	2.31
Serine	0.96	2.30	1.93
Tyrosine	0.71	2.00	1.52
Total amino acids	21.92	61.55	43.09
Chemically available lysine	1.66	4.86	2.64
Lysine:CP ratio, %	6.91	7.60	6.31
Trypsin inhibitor activity, mg/g	1.70	—	N/A^1^

^1^N/A, not analyzed.

### Supplemental protein source

The AID of GE and DM was lower (*P* < 0.001; Table 4) feeding the SBM than FM diets. The ATTD of GE and DM, DE value, and AHF of CP did not differ between the SBM and FM diets, but the ATTD of CP was greater for FM than SBM diets. The AHF of GE and DM was greater (*P* < 0.05) feeding the SBM than FM diets. The AID of CP and all AA except methionine was greater (*P* < 0.001; Table 5) feeding the FM than SBM diets.

**Table 4.** Apparent ileal digestibility (AID), apparent total tract digestibility (ATTD) and apparent hindgut fermentation (AHF) of crude protein, dry matter, gross energy, and digestible energy of experimental diets containing supplemental plant or animal protein with or without extrusion

	Supplemental plant protein		Supplemental animal protein			*P*-value		
Item	Mash	Extruded	Mash	Extruded	SEM^1^	Supplemental protein	Extrusion	Supplemental protein × extrusion
AID, %								
Dry matter	62.2	62.9	66.5	67.7	0.98	< 0.001	0.179	0.707
Gross energy	64.0	65.1	68.0	69.9	0.97	< 0.001	0.054	0.586
ATTD, %								
Dry matter^2^	82.7	83.0	82.0	82.6	0.45	0.088	0.202	0.601
Gross energy	81.3	82.7	81.5	83.5	0.49	0.165	< 0.001	0.449
Crude protein	79.9	81.2	82.7	84.7	0.67	< 0.001	0.024	0.636
AHF^3^, %								
Dry matter	20.5	20.1	15.5	14.9	0.88	< 0.001	0.394	0.880
Gross energy	17.3	17.6	13.5	13.6	0.92	< 0.001	0.791	0.867
Crude protein	4.7	3.3	5.9	5.7	1.46	0.065	0.436	0.441
DE, Mcal/kg as fed	3.26	3.33	3.21	3.34	0.02	0.131	< 0.001	0.094

^1^Least-squares means based on 8 observations per diet.

^2^For ATTD of DM a carryover effect was detected, but not for any of the other variables.

^3^AHF = ATTD—AID.

**Table 5.** Apparent ileal digestibility of crude protein and amino acids of experimental diets containing supplemental plant or animal protein with or without extrusion

	Supplemental plant protein		Supplemental animal protein			*P*-value		
Item, %	Mash	Extruded	Mash	Extruded	SEM^1^	Supplemental protein	Extrusion	Supplemental protein × Extrusion
Crude protein	75.2	77.9	76.8	79.0	0.81	0.029	<0.001	0.671
Indispensable amino acids								
Arginine	81.9	84.8	84.4	86.7	0.74	<0.001	<0.001	0.552
Histidine	77.0	78.0	79.0	80.5	1.01	<0.001	0.091	0.726
Isoleucine	75.5	79.9	79.2	83.6	0.87	<0.001	<0.001	0.974
Leucine	75.5	79.9	79.6	83.7	0.94	<0.001	<0.001	0.812
Lysine	75.4	76.9	78.7	80.6	1.03	<0.001	0.026	0.799
Methionine	82.9	85.3	84.1	85.7	0.76	0.146	0.001	0.500
Phenylalanine	75.1	80.5	78.6	83.5	0.93	<0.001	<0.001	0.739
Threonine	70.4	73.6	73.8	78.4	1.03	<0.001	<0.001	0.374
Tryptophan	78.3	80.4	80.5	85.1	1.26	<0.001	0.001	0.182
Valine	73.0	76.7	76.4	80.3	1.01	<0.001	<0.001	0.913
Dispensable amino acids								
Alanine	70.7	75.8	76.4	79.6	1.07	<0.001	<0.001	0.248
Aspartic acid	74.7	77.6	76.6	79.7	0.90	<0.001	<0.001	0.876
Cysteine	65.7	63.0	69.7	68.5	1.48	<0.001	0.066	0.453
Glutamic acid	82.0	83.3	84.6	86.3	1.05	0.001	0.058	0.799
Glycine	65.3	66.7	73.0	71.4	1.47	<0.001	0.939	0.161
Proline	73.9	77.8	77.8	80.5	1.02	<0.001	<0.001	0.443
Serine	73.6	77.6	76.2	79.5	0.91	0.001	<0.001	0.550
Tyrosine	76.2	80.4	78.6	83.4	0.80	<0.001	<0.001	0.619

^1^Least-squares means based on eight observations per diet.

### Extrusion

Interactions between supplemental protein source and extrusion were not observed for the analyzed response variables. Extrusion did not affect the AID of DM and GE, the ATTD of DM, or the AHF of DM, GE, and CP (Table 4). Extrusion increased (*P* < 0.001) the ATTD of GE and CP, and DE values feeding both the SBM and FM diets. Extrusion increased (*P* < 0.05; Table 5) the AID of CP and most AA except for histidine, cysteine, glutamic acid, and glycine feeding the SBM and FM diets.

## DISCUSSION

### Lentil Grain

Lentil not used for human consumption can be an important energy and protein source in swine nutrition (Landero et al., 2012; Woyengo et al., 2014; Hugman et al., 2021a). In the present study, the analyzed content of dietary fiber, starch, CP, and AA in lentil was similar to previously reported values (Landero et al., 2012; NRC, 2012; Woyengo et al., 2014; Hugman et al., 2021a). Lentil contains relatively high fiber that is mostly insoluble, namely ADF and lignin (Vasishtha and Srivastava, 2011; Srivastava and Vasishtha, 2013), which is associated with low utilization and digestibility of energy and AA in pigs (Souffrant, 2001; Lindberg, 2014). Lentil contains ANF such as protease inhibitors, lectins, and tannins that may affect growth performance and metabolic processes in pigs (Van Heugten, 2001; Landero et al., 2012). Thus, feed processing technologies to increase nutrient availability and digestibility and the feeding value of lentil-based diets are important (Hugman et al., 2021a).

### Supplemental Protein Source

Animal proteins such as FM are used as supplemental animal protein in diets for pigs as readily digestible protein with a high content of indispensable AA and without the detrimental effects of ANF, fiber, and phytate (Kim and Easter, 2001). Furthermore, growth performance of weaned pigs fed diets containing FM was increased and their immune function improved (Newport and Keal, 1983; Kim and Easter, 2001). Plant proteins such as SBM can be a cost-effective alternative to animal proteins because FM can be three times more expensive than SBM (Jeong et al., 2016). However, SBM contains ANF that can limit feed intake and nutrient utilization (Woyengo et al., 2017). Further processing of SBM can reduce the levels of ANF (Long et al., 2015; Hugman et al., 2021a). Additionally, compared to animal proteins, plant protein ingredients contain cell walls and dietary fibers that may reduce the rate of nutrient digestion and absorption (Wu et al., 2018).

The reduced AID of DM, GE, and CP in the SBM diet can be attributed to the SBM diet containing more ADF and ANF than the FM diet. Furthermore, the lower digestibility of nutrients in pigs fed SBM could also be attributed to the presence of indigestible carbohydrate complexes and proteins such as glycinin and β-conglycinin that can interrupt nutrient digestion and absorption (Li et al., 1991; Chen et al., 2021). The ileal digestibility of GE and CP was lower in pigs fed SBM as compared with fermented SBM, whey protein concentrate, and FM in previous studies (Yun et al., 2005; Yan et al., 2022). However, the DE value did not differ between diets in the present study, likely because hindgut fermentation of DM and GE was greater for the SBM than FM diet. Fiber in SBM may contribute dietary energy for pigs via hindgut fermentation (Woyengo et al., 2016). Indeed, feeding SBM diet increased AHF of DM and GE, likely due to SBM containing 14% more ADF and having a lower AID of GE than the FM diet, which could be explained by more fiber entering the large intestine for fermentation (Carneiro et al., 2008). Therefore, the increased DM and GE fermentability of the SBM diet coincides with its lower AID of nutrients.

### Extrusion

Extrusion may be applied to individual ingredients or complete diets (Rojas and Stein, 2017). This processing technology involves initially steam conditioning followed by buildup of friction pressure within the extruder barrel and provision of additional heat along the barrel of the extruder (Hancock and Behnke, 2001). Extrusion may therefore increase the solubility of dietary fiber, denature protein, and reduce ANF in canola meal, field pea, or lentil grain (Heyer et al., 2021; Hugman et al., 2021a). However, in the present study, extrusion did not noticeably alter the concentration of macronutrients in diets except for dietary crude fat, indicating that extrusion settings used to process these diets did not affect cell wall structure sufficiently. The observed reduced crude fat content in extruded than mash diets might have been caused by the processing conditions of extrusion that affected lipid stability (Cargo-Froom et al., 2023). High temperatures during extrusion can induce the formation of lipid-amylose complexes that may not be analyzed as crude fat (Cervantes-Ramírez et al., 2020).

Extrusion is a thermomechanical process that can modify structures, potentially opening up the cell wall structure and thereby increase fiber digestibility that may increase the nutritional value of individual ingredients or complete diets (Rojas and Stein, 2017; Heyer et al., 2021). Insoluble fiber can represent up to 99.7% of the total dietary fiber in lentil (De Almeida Costa et al., 2006). Cellulose, hemicellulose, and lignin, the major constituents of total dietary fiber in lentil (Khan et al., 2001), cannot be degraded by the pig endogenous enzymes, but can be degraded to a certain extent by microbiota in the hindgut. Extrusion increased ATTD of GE but did not increase the digestibility of DM, AID of GE and AHF of GE, CP, and DM, which could be explained by a lack of substantial physicochemical changes of most nutrients in extruded vs. mash diets. Furthermore, the lack of differences in digestibility and fermentability of nutrients in the present study coincides with the observed lack of differences in concentrations of energy and nutrients between extruded and mash diets. In contrast, extrusion of a corn-SBM diet increased the AID of DM and GE (Rojas et al., 2016). The difference in response could be due to differences in type of crystalline structure in starch granules in corn and lentil, and the greater starch content in a corn-based diet that will become better digestible if extruded, thereby increasing nutrient and energy digestibility (Tan et al., 2021).

Extrusion increased ATTD of CP of lentil-grain based diets in the present study, in agreement of extrusion of solely lentil increasing ATTD of CP while simultaneously reducing lentil TIA (Hugman et al., 2021a). Extrusion might increase CP digestibility by reducing TIA in lentil. Trypsin inhibitor compounds can increase endogenous losses of AA and inhibit access of endogenous enzymes to proteins, thereby reducing CATTD of CP (Jezierny et al., 2010). In contrast, extrusion of field pea did not affect diet ATTD of CP in weaned pigs (Hugman et al., 2021b). In a recent study, extrusion did not affect the ATTD of CP of canola meal in 70-kg pigs (Heyer et al., 2021). Increased DE value in extruded diets was not caused by increased fermentation of nutrients as indicated by the lack of differences in AHF of DM, GE, and CP. Instead, the increased ATTD of GE in extruded diets was likely initiated in the small intestine and a result of increased AID of CP and AA. Indeed, extrusion tended to increase AID of GE in the present study. The results are similar to extrusion increasing ATTD of GE of lentil and field pea (Hugman et al., 2021a, 2021b). Based on the inconsistent effects of extrusion on nutrient digestibility obtained in the present study, we conclude that extrusion did not disrupt the cell wall structure of dietary ingredients sufficiently.

Hydrothermal treatments can partly denature proteins and disrupt the fiber matrix of ingredients and diets (Svihus and Zimonja, 2011; Salazar-Villanea et al., 2016; Rojas and Stein, 2017). The disruption may render more protein susceptible to intestinal proteases, thereby increasing AA digestibility (de Vries et al., 2012). Previously, applying heat treatment to corn, wheat, and barley-based diets containing SBM, DDGS, soybean hulls, or cassava increased AID of CP and indispensable AA (Vande Ginste and De Schrijver, 1998; Rojas et al., 2016). Extrusion of diets increased AID of CP and most indispensable AA in the present study. The increased AID of AA observed in the present study indicates that the extruded diets contained more dietary AA than the mash diets. Therefore, animal protein sources that are often expensive could be replaced by plant-based, cost-effective alternatives, such as extruded SBM, thereby fully or partially offsetting the cost of extrusion.

In conclusion, diets containing fish meal as supplemental protein had greater ileal digestibility of DM, energy, CP, and most AA than diets containing soybean meal as supplemental protein. Extrusion increased the AID of CP and most AA, and DE value of lentil-grain based diets containing either supplemental plant or animal protein, indicating that extrusion is effective in increasing the nutritional value of lentil-grain based diets fed to growing pigs.
